# The use of light spectrum blocking films to reduce populations of *Drosophila suzukii* Matsumura in fruit crops

**DOI:** 10.1038/s41598-020-72074-8

**Published:** 2020-09-21

**Authors:** Michelle T. Fountain, Amir Badiee, Sebastian Hemer, Alvaro Delgado, Michael Mangan, Colin Dowding, Frederick Davis, Simon Pearson

**Affiliations:** 1 NIAB EMR, New Road, East Malling, Kent, ME19 6BJ UK; 2 School of Engineering, University of Lincoln, Brayford Pool, Lincoln, LN6 7TS UK; 3 Berry Garden Growers, Tatlingbury Oast, Tonbridge, Kent, TN12 6RG UK; 4 School of Computer Science, University of Lincoln, Brayford Pool, Lincoln, LN6 7TS UK; 5 Department of Computer Science, University of Sheffield, Regent Court, Sheffield, S1 4DP UK; 6 Lincoln Institute for Agri-Food Technology, University of Lincoln, Riseholme Campus, Lincoln, LN2 2LG UK; 7 Department of Chemistry, University of Reading, Whiteknights, RG6 6AD UK

**Keywords:** Biological techniques, Biophysics, Plant sciences, Zoology, Ecology, Environmental sciences, Materials science

## Abstract

Spotted wing drosophila, *Drosophila suzukii*, is a serious invasive pest impacting the production of multiple fruit crops, including soft and stone fruits such as strawberries, raspberries and cherries. Effective control is challenging and reliant on integrated pest management which includes the use of an ever decreasing number of approved insecticides. New means to reduce the impact of this pest that can be integrated into control strategies are urgently required. In many production regions, including the UK, soft fruit are typically grown inside tunnels clad with polyethylene based materials. These can be modified to filter specific wavebands of light. We investigated whether targeted spectral modifications to cladding materials that disrupt insect vision could reduce the incidence of *D. suzukii*. We present a novel approach that starts from a neuroscientific investigation of insect sensory systems and ends with infield testing of new cladding materials inspired by the biological data. We show *D. suzukii* are predominantly sensitive to wavelengths below 405 nm (ultraviolet) and above 565 nm (orange & red) and that targeted blocking of lower wavebands (up to 430 nm) using light restricting materials reduces pest populations up to 73% in field trials.

## Introduction

*Drosophila suzukii* Matsumura (spotted winged drosophila, SWD) is an invasive pest responsible for significant losses in global supply of soft and stone fruits^1^. Damage is amplified as *D. suzukii* lays eggs in un-ripened fruit^2^. Economic impacts are significant; losses from large scale infestation (20% loss) across the US alone could equate to farm gate impacts > $500M^3^. *D. suzukii* has challenged existing Integrated Pest Management (IPM) methodology since controls used to protect fruit rely on frequent applications of plant protection products^4^. The pest, which has multiple generations in a season, is showing reduced sensitivity to some insecticides^5,6^ . In addition, insecticide applications are not only undesirable but can have a detrimental influence on biological control agents^7^. Furthermore, the use of insecticides for *D. suzukii* are rightly restricted to reduce environmental impact and residues in fresh produce.

Approaches which can reduce or interfere with the *D. suzukii* lifecycle are needed to reduce disruption to IPM strategies and ultimately damage to fruit. Most commonly this is done using olfactory cues, for example, sex or aggregation pheromones which attract pests into a trap or away from the crop^8^. Currently, mass traps using non-species-specific fermenting baits are employed around crops to reduce populations in wild habitats but these are not effective within the crop once it is fruiting^9^. Another approach might be to interfere with visual cues via spectral modifications to greenhouse cladding materials. This approach has been deployed to modify plant habit^10,11^, reduce the impact of fungal pathogens^12^ and insect pests, typically in the Hemipitera and Thysanoptera orders^13,14^. To date, no greenhouse cladding material has been optimised to interfere with Diptera order vision; although observational studies suggests that removing UV light may reduce impacts from unidentified Dipterans in cucumber^15^ and red amaranth crops^16^. In particular, no studies have optimised the effects of UV attenuating claddings on *Drosophila* species, including *D. suzukii*^14^.

The vision systems of the related species, *D. melanogaster*, are well defined. *D. melanogaster* have compound eyes with five spectral sensitivities, each with c. 700 ommatidia^17^. Each ommatidium contains three families of photoreceptors; R1-R6, R7 and R8. All three photoreceptor sets are thought to be involved in both motion detection and colour vision^18–20^. Spectral sensitivity for motion shows two peaks, one at c.350 nm and a second at c.470 to 500 nm^21,22^. In addition, *D. melanogaster* are highly sensitized to polarised light and use this for flight navigation^23^ Spectral quality has complex impacts on insect behaviour, whilst *D. melanogaster* orientate towards UV light they have been shown to have an aversion to egg laying at the same wavebands^24^. Likewise, insect larvae show an aversion to UV light^25^.

Hitherto, the spectral sensitivity of *D. suzukii* has not been explored, although it is considered vision between *Drosophila spp* is highly conserved^26^. Colour perception studies on *D. suzukii* have focussed upon the optimisation of coloured insect traps. These showed that the insects were attracted preferentially to red and black traps compared to other colours (purple, orange, green, yellow, blue and white)^27,28^. Additionally colour contrast (background colour) rather than appearance is critical to attraction^28,29^.

The aim of this study was to establish whether disruption of insect vision by spectral filtering of specific wavebands of light could reduce *D. suzukii* numbers in the cropping area and fruit damage impact. We tested the efficacy of a range of wavelengths through laboratory controlled choice tests, small cloche and then field scale experiments. Through the use of insect behavioural responses to spectral waveband, material design, and field tests, this work demonstrated that novel spectral filters can contribute to the control of a serious invasive fruit crop pest.

## Results

### Laboratory choice tests

Initial laboratory choice tests investigated the spectral sensitivity of *D. suzukii* before comparing their spectral preferences. *D. suzukii* were given a choice of two visual stimuli (light of different spectra or dark controls). The ratio of insects choosing to approach each stimulus after 30 min exposure was used to define the attraction index (AI). AIs of 1 or -1 would indicate that all individuals approached either source, whereas an AI of 0 indicates an equal preference.

**Figure 1:**
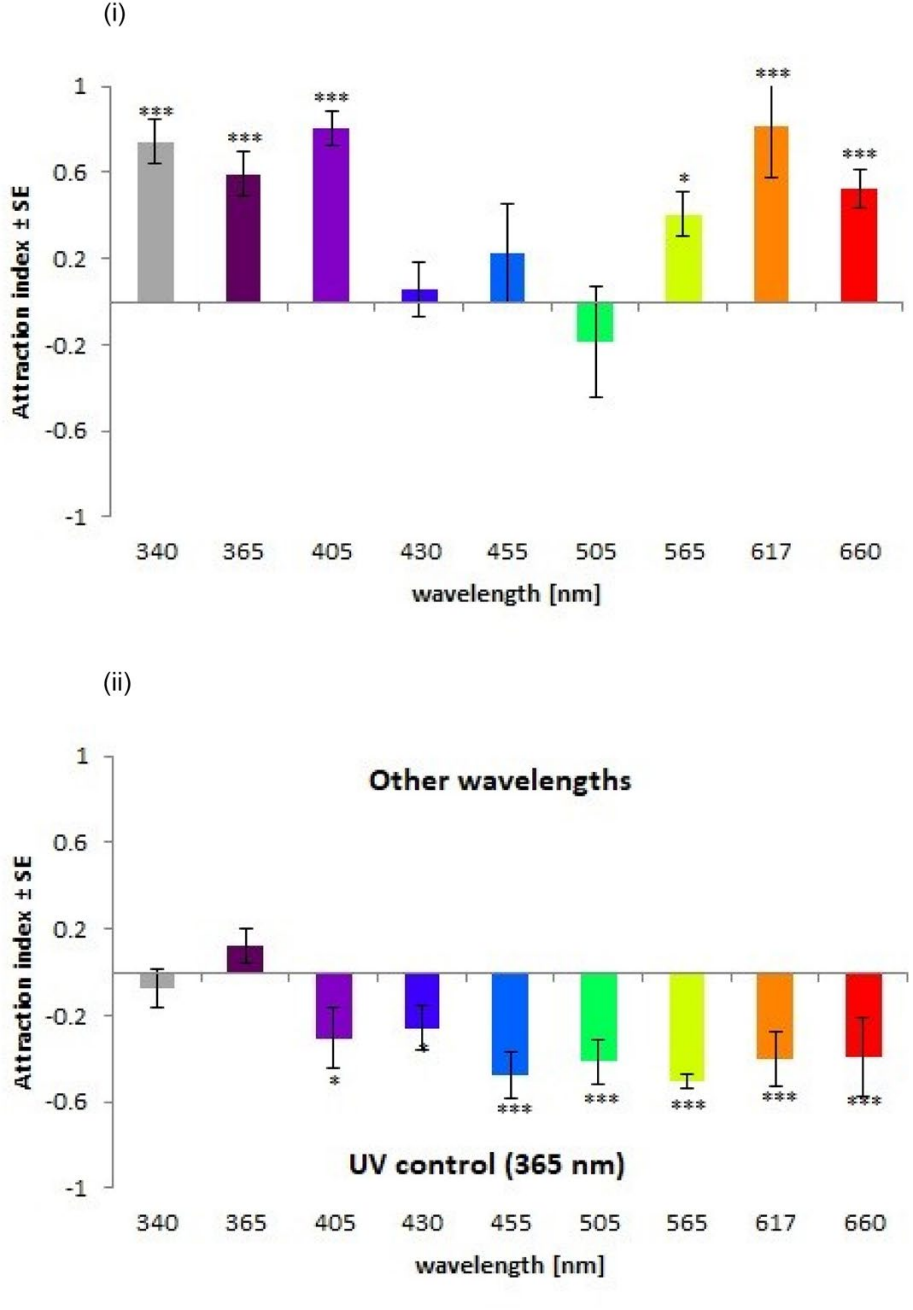
**(i**) Spectral sensitivity tests (dark vs waveband): Mean (± S.E) attraction indices (A.I.) of female *D. suzukii* to identical light treatments in both arms of a laboratory Y-maze (bias tests). (ii) Preferential choice tests (365 nm vs waveband): Mean (± S.E) attraction indices (A.I.) of female *D. suzukii* to different wavelengths versus dark. Asterisks above bars indicate a less than *** < 0.001, ** < 0.01, * < 0.05 probability where flies made a choice between Ymaze arms. (A.I. was calculated from the observed total proportion of flies in each arm, summed from all replicate T-maze tests and calculated using the binominal distribution assuming an underlying 1:1 proportion).

To reveal spectral sensitivity flies chose between a dark stimulus and a set waveband of light (Table 3, 340 to 660 nm)with perceptible light triggering the animals innate phototactic approach behaviour^18^. *D. suzukii* females preferentially approached light in the near-UV range (340, 365, 405 nm) in common with *D. melonagastor*^30^ that possess UV-sensitive photoreceptor R7p and R7y and true colour vision with photoreceptor rhodopsins Rh3 and Rh4^31–33^. However, *D. suzukii* females did not preferentially approach light in the 430–565 nm range as would be predicted possessing similar blue-green sensitive RX and RY photoreceptors as *D. melonagastor* but rather show preferences for orange and red light (617 and 660 nm), raising the potential for divergence in spectral sensitivities between species (Fig. 1i).

Then differential phototaxis experiments^18^ were conducted which tested wavelength preference between the discrete wavebands listed above and a 365 nm control (Table 3). No significant preference was observed between 365 and 340 nm stimuli but *D. suzukii* showed a preference for 365 nm when presented in combination with higher wavebands (405 nm-660 nm) (Fig. 1ii). The strength of the effect increased from AI’s of − 0.25 and − 0.3 (p < 0.05) for lower wavebands representing violet (405 nm) and blue light (430 nm), to a consistent c.-0.5 (p < 0.01) for all higher wavebands. In summary, *D. suzukii* showed a differential and preferential phototactic response to ultra-violet light over visible light.

### Semi-field "no-choice" fruit damage experiments

A hypothesis was then tested to investigate whether removing ultra-violet radiation in the terrestrial environment via spectral filters applied to greenhouse cladding materials could reduce pest impacts.

**Figure 2:**
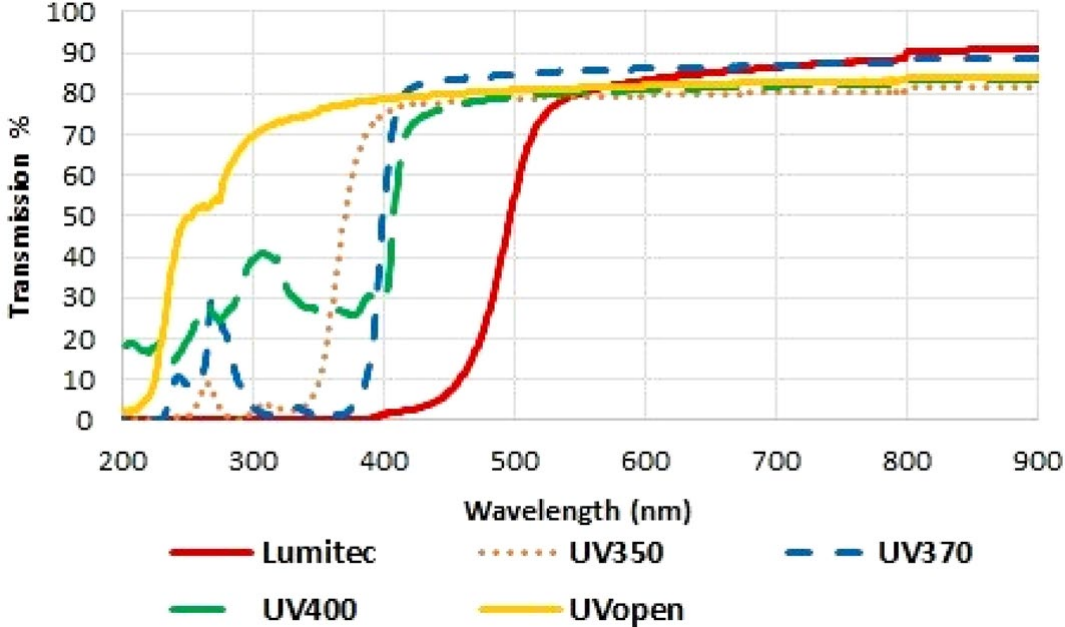
Spectral transmission (%) versus wavelength (nm) for all cladding films measured by Cary 7,000-Diffuse using Reflectance Accessories.

Spectral transmissions of the materials are shown in Fig. 2; experimental materials absorbed UV up to and between 350 to 430 nm.

In the first year (2016) semi-field experiments with strawberry crops, the effect of cladding absorbing up to 350 nm of UV against an "open" control cladding on *D. suzukii* emergence from strawberry fruit was tested. In addition, the impact of clear versus highly diffuse materials which scattered a significant proportion of inbound radiation was also tested. These experiments showed there were no significant differences between the UV light transmission clear or diffuse cladding in the umbers of *D. suzukii* which emerged per gram of fruit; UV light transmission (F [1, 6] = 4.6, p = 0.076), clear or diffuse films (F [1, 6] = 0.5, p = 0.839). No significant difference was observed over time (F [2, 15] = 0.43, p = 0.573) and there were no interactions (time.treat: F [2, 15] = 3.32, p = 0.094; time.film: F [2, 15] = 0.02, p = 0.938; time.treat.film: F [2, 15] = 1.01, p = 0.362). Although differences between treatments were not significant, the UV350 film had fewer *D. suzukii* per gram of fruit than the open control.

In 2017, the tests were extended to compare three materials with different UV absorbance cut-offs against the open control. Tests which included UV370 and UV400 did not result in significantly fewer *D. suzukii* emerging per gram of fruit (F [3, 6] = 1.96, p = 0.221) compared to the control (Table1). Once more, the date fruit was sampled, was significant for *D. suzukii* emerging per gram (F [2, 16] = 8.9, p = 0.008). There was no interaction between fruit sampling time and cladding treatment (F [6, 16] = 0.28, p = 0.893).

**Table 1:** Numbers of adult *D. suzukii* emerging per gram of strawberry fruit from tunnels clad with different UV-attenuating films in 2016 and 2017 (mean (± S.E)).

Year	Treatment	Film	Per gram
2016	UVopen	Clear	0.41 ± 0.17
		Diffuse	0.44 ± 0.19
	UV350	Clear	0.10 ± 0.04
		Diffuse	0.09 ± 0.02
2017	UVopen	Clear	0.04 ± 0.01
	UV350	Clear	0.05 ± 0.03
	UV370	Clear	0.04 ± 0.02
	UV400	Clear	0.02 ± 0.01

There was no significant difference between cladding treatments in either year, although there was a suggestion of decreasing *D. suzukii* with increasing UV attenuation.

**Figure 3:**
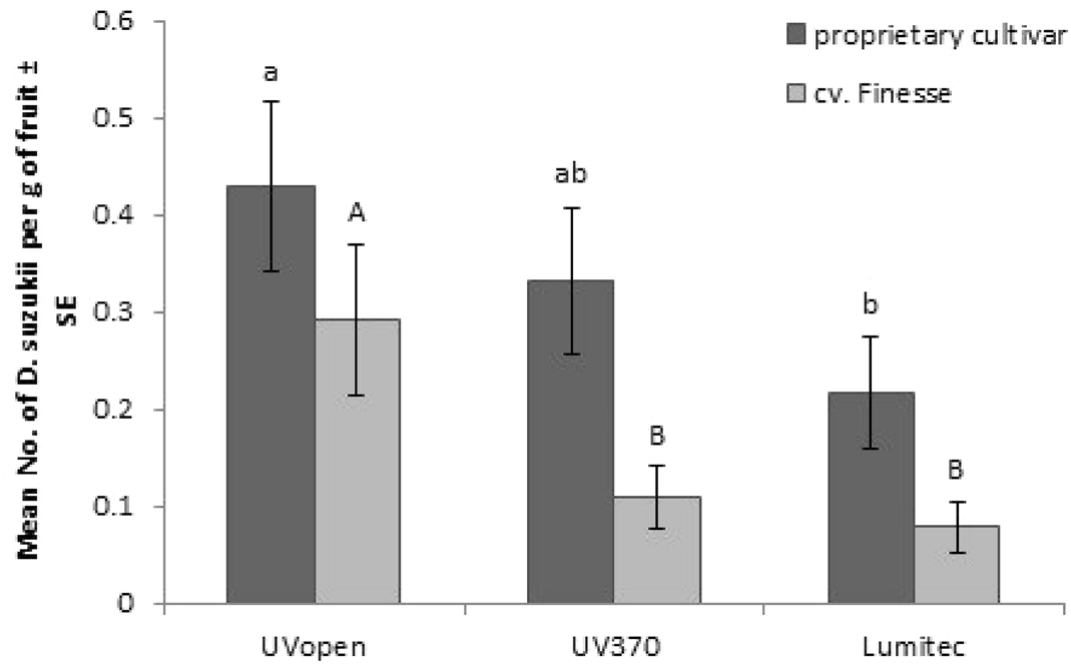
Mean (± S.E) numbers of *D. suzukii* emerging per gram of strawberry from tunnels clad with UV-attenuating films compared to a UV open control, in 2018. Different letters (lower-case within the proprietary cultivar, capital within cv. Finesse) denote significant differences between films (Fisher’s LSD, *α* = 0.05).

The 2018 experiments compared a material with a UV cut off up to 430 nm (Lumitec) against the control cladding and the UV370 material deployed in 2017. *D. suzukii* numbers emerging from fruit were significantly higher under UVopen compared to UV attenuating claddings (UVtransmission: F [2, 35] = 10.91, p < 0.001, variety: F [1, 35] = 21.19, p < 0.001, UVtransmission.variety: (F [2, 35] = 0.59, p = 0.559, time: (F[3, [126] = 31.49, p < 0.001, time.UVtransmission: F[6, 126] = 2.23, p = 0.057, time.variety: F[3, 126] = 156.83, p < 0.001, and time.UVtransmission.variety: F[6, 126] = 4.78, p < 0.001; (Fig. 3)).

**Figure 4:**
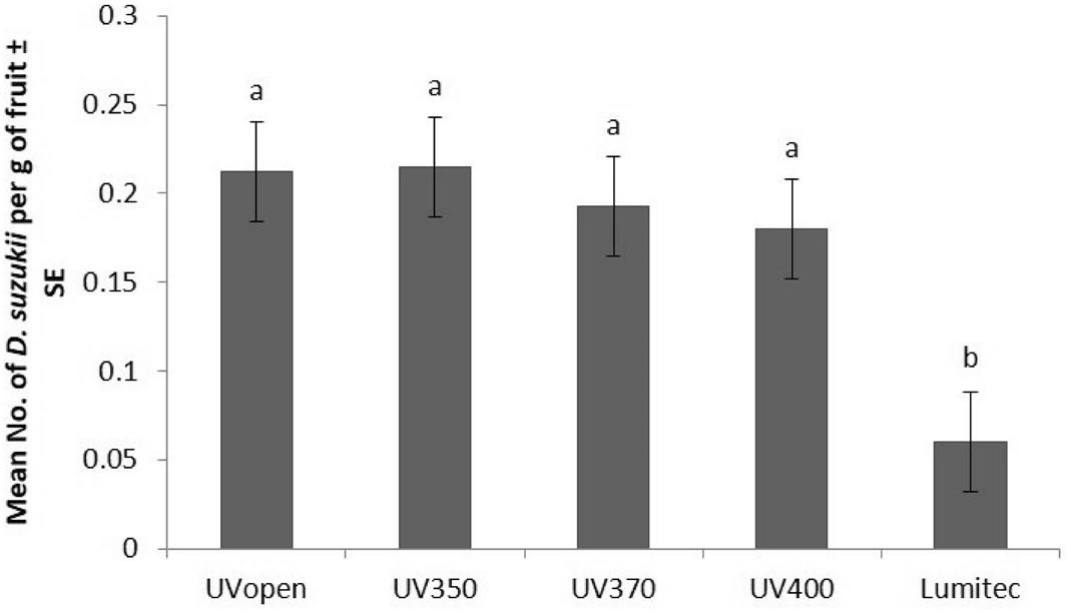
Predicted mean (± S.E) numbers (flies per gram of fruit) of *D. suzukii* under UV-attenuating claddings (n_UVopen_ = 82, n_UV350_ = 18, n_UV370_ = 73, n_UV400_ = 9, n_UV430_ = 64). Different letters denote significant differences between films (Tukey’s HSD, α = 0.05). The error bars depict the average standard error of differences between the pairs.

Statistical analyses of the three-year combined data set showed significant differences between the UV-attenuating claddings for *D. suzukii* emergence per gram of fruit (F[4,38.7] = 4.4, p = 0.005). Adult *D. suzukii* emergence was reduced up to 8%, 22%, 34%, and 73% for UV350, UV370, UV400, and Lumitec claddings respectively, compared to the UVopen cladding (Fig. 4).

### Cloche choice experiments

**Figure 5:**
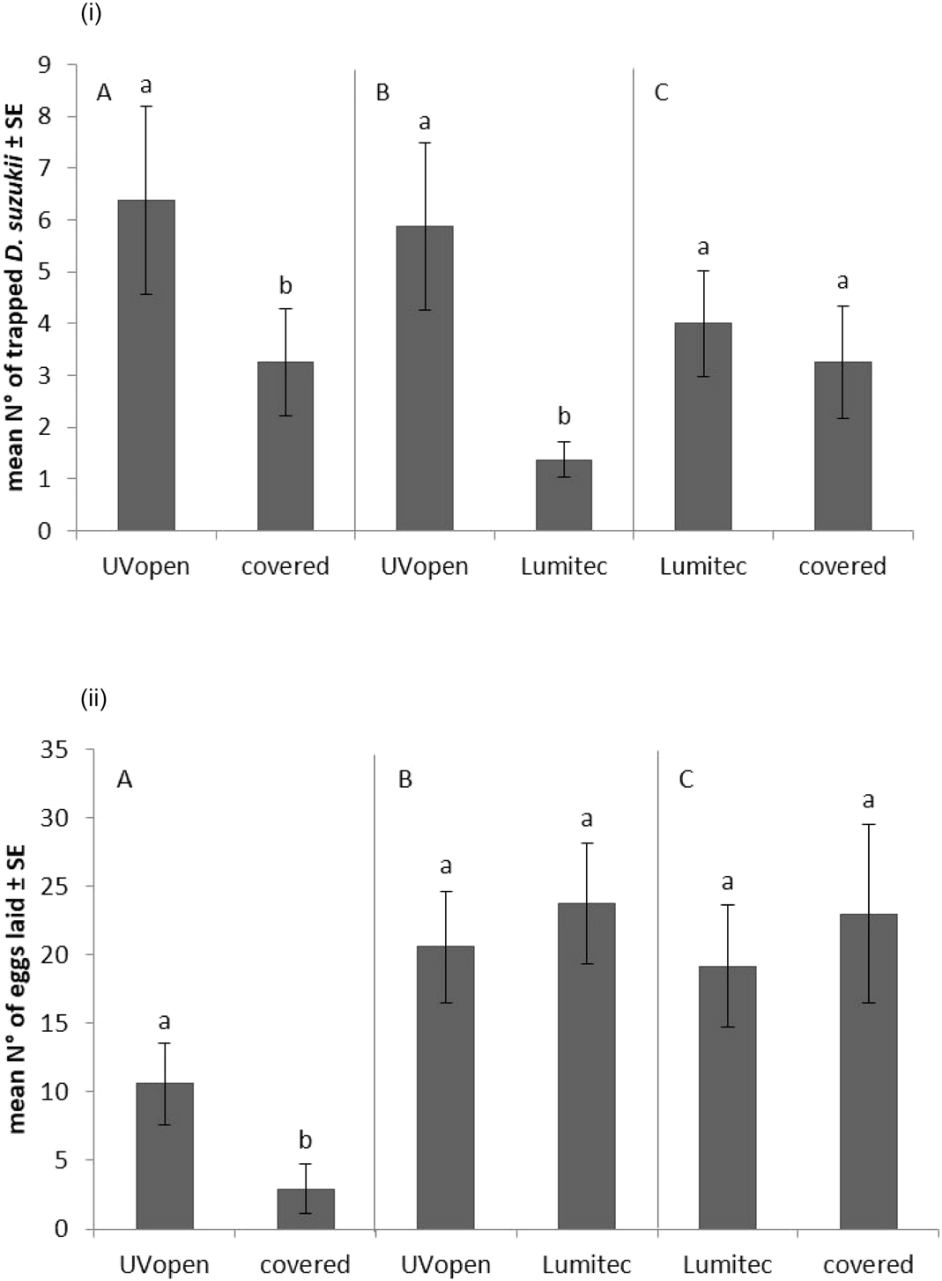
(**i**) Mean numbers (± S.E) of female *D. suzukii* recovered on yellow sticky traps (no fruit) from different experiments (**A**–**C**) in the two-way choice cloche experiment with UV430-attenuating film in comparison to covered (dark) and UVopenfilm. Different letters denote significant differences between cladding materials (Tukey’s HSD, α = 0.05). (**ii**) Mean (± S.E) numbers of *D. suzukii* eggs laid in blueberry fruits in different experiments (**A**–**C**) in the two-way choice cloche tests withUV430-attenuating cladding materials in comparison to covered (dark) and UVopen cladding. Different letters denote significant differences between cladding materials (Tukey’s HSD, α = 0.05).

In cloches with no fruits, the numbers of adult *D. suzukii* entering the "open" cloches compared to the light excluded or Lumitec cladding were significantly higher (t = 2.38, df = 7, p = 0.049, and t = 2.90, df = 7, p = 0.023, Fig. 5i, respectively). There was no difference in the numbers of *D. suzukii* captured under the Lumitec and light exclusion claddings (t = 1.18, df = 7, p = 0.276, Fig. 5i).

In choice experiments between two cloche compartments, both containing blueberry fruit in a Petri dish (no plant foliage), but with different cladding materials, *D. suzukii* preferred to lay eggs under UVopen film compared to the light exclusion (dark) cloche (t = 6.34, df = 9, p < 0.001, Fig. 5ii). There was no preference when UVopen and Lumitec (absorbs up to c.430 nm) were compared (t = 0.58, df = 9, p = 0.575), nor Lumitec and light exclusion (t = 0.1, df = 9, p = 0.923, Fig. 5ii). The generally increased oviposition in UVopen vs. Lumitec, and Lumitec vs. the covered/dark cloche (Fig. 5ii) was noteworthy.

## Discussion

### Summary of key results

These experiments show, for the first time, a clear relationship between the level of UV attenuation in greenhouse claddings and the attraction (demonstrated in the laboratory Y-maze and cloche choice experiments) and ultimate oviposition (demonstrated in the cloche and semi-field experiments) of *D. suzukii*.

Laboratory choice experiments investigated the phototactic behaviour of *D. suzukii* females to wavelengths from 340–660 nm showing positive phototactic responses to light in the wavebands 340–405 nm (UV) and 617- 660 nm (orange & red), but not in the range of 430–565 nm (blue & green). Sensitivity to UV light is found in many insects^34^ with clear benefits for sky detection^35^ which, when combined with a positive phototactic reflex, will naturally guide individuals to the outdoors. This hypothesis is supported by the data from cloche experiments in which *D. suzukii* females preferentially entered tunnels illuminated with UV (and near visible) light over those with the same spectra attenuated. It is noted however that attraction to red/orange and not blue/green light diverges from the predictions of a highly conserved spectral sensitivity across fruit-fly species^26^ with *D. melanogaster* sensitivity decreasing above > c.480 nm^21,22^. It is fascinating to consider whether *D. suzukii* have evolved away from the common fruit-fly not just in its ovipositor function but also in its visual sensing with likely adaptations to improve visual fruit detection. Neuroanatomical analysis of the characteristics of the photoreceptors present in *D. suzukii’s* eye present an obvious research avenue.

In differential phototaxis experiments, *D. suzukii* showed a preference towards UV light (365 nm) over higher wavelengths (405–430 nm: weak preference; 455–660 nm: strong preference). It is therefore clear that the need to remain outdoors (expressed by a preference for UV light) is dominant in *D. suzukii*, but a secondary attraction to wavelengths (orange and red) that provide a possible secondary mechanism allowing for robust visual fruit detection was also noticed. Colour-opponency is thought to underpin colour perception in fruit flies, and has recently been used to enhance the performance of artificial fruit detectors^36^. Computational modelling of the sensory and neural perception systems facilitating fruit detection in insects will allow evermore targeted and nuanced interventions and provides an excellent channel for future investigation.

Short term choice experiments in field cloches showed adult females were more likely to enter the UVOpen treatment compared to Lumitec (430 nm absorption), however, there was no difference in numbers of eggs laid between treatments. These effects are not surprising since *Drosophila spp.* have complex responses to UV, whilst orientation has a preferential response to UV, females have an aversion to laying eggs under UV^24^. In addition, cues other than phototaxis could play a role, for example, olfactory responses have a significant role in fruit localisation by *Drosophila spp.*^37,38^. The complexity and contradictory nature of these responses to UV challenges whether the use of UV absorbing materials can support efficacious control of the pest in greenhouse crops. Net effects of UV on insect populations can only be established through longer term projects over multiple lifecycles.

Here, net effects were tested in "no choice" experiments conducted over a full production season. Although the magnitude of the response of *D. suzukii* to lower wavelengths of UV-attenuating claddings was not significant in the low replicated (× 3) no-choice experiments in the first two years the trend was encouraging and following experiments showed a significant reduction in *D. suzukii* in fruit with increasing UV-attenuation in experiments with higher UV-attenuation and increased replication (× 8). These showed *D. suzukii* egg laying decreased significantly with increasing attenuation of light transmission up to 430 nm (maximum tested). Numbers of eggs laid (measured by adult *D. suzukii* emergence from fruit) were reduced up to 8%, 22%, 34%, and 73% for UV350, UV370, UV400, and Lumitec (absorbs up to 430 nm) respectively, compared to the UVopen claddings. These experiments confirm the impact of UV attenuation over multiple insect lifecycles and suggest that the approach can be used to suppress population and infestation pressure. *D. suzukii* were less likely to reproduce in areas under UV attenuation of 430 nm even in the presence of a fully cropping field strawberries which resulted in less fruit damage and losses of yield. These impacts are of commercial significance to fruit farmers but additional work is required to quantify the mechanisms driving the response. It was shown that short-term variance in UV/blue light can impact fly orientation including egg laying aversion but longer-term mechanisms have not been fully explored.

This is the first time that UV-attenuating claddings have shown efficacy for contributing to the control of *D. suzukii* and the first study conducted using strawberry^14^. In Fennell et al. (2019)^14^ the main mechanisms of cladding suppression of insect pests were considered to be (i) positive phototaxis to ultraviolet light sources, and (ii) reduced take-off and flight behaviour when UV was absent. Suppression of other common pests including thrips^13,39–41^ and aphids^13,15,39,42–53^ are attributed to both a reduction in pest immigration into the crop^15^, and within crop movement^54^. In our field crop studies, *D. suzukii* was artificially introduced into the cropping area demonstrating a within-crop effect. In addition, the cloche experiments relied on *D. suzukii* immigrating into the cladded areas. The latter was not affected by the claddings when fruit was available indicating that the primary driver when given no choice was to orientate to fruit—most likely with olfactory cues associated with both fruit compounds and associated fermenting yeasts^37,55^ .

The claddings appear to be interfering with host location and subsequent egg laying. In the blueberry cloche experiment where fruits were introduced on a Petri dish and not with background plant foliage, *D. suzukii* successfully laid eggs. It is possible that *D. suzukii* locates fruit visually by colour contrast (with plant foliage) as suggested by Little et al. (2018)^29^ rather than colour appearance. From their experiments *D. suzukii* had a limited ability to comprehend red. Female *D. suzukii* were attracted to purple sticky discs (5 cm) on a white background^29^. In another similar recent study red, purple, and black disks were more attractive when presented against a white background. Male and female *D. suzukii* responded identically in these tests. Significantly more male and female *D. suzukii* were captured on the red and yellow disks than those presenting the corresponding grayscale for that colour^28^. Hence, background contrast to fruits is important in fruit recognition and it is possible that claddings used in this work interfered with this contrast and hence orientation to fruits within the strawberry crop, but not in blueberries placed on the ground on a Petri dish.

Potentially a more effective strategy would be to incorporate the claddings as part of an integrated pest management approach including repellents, to further inhibit *D. suzukii* entering crops^56,57^, and attractants where semiochemical based baits are placed outside the crop to intercept and further reduce immigration into the cropping area^58^. This could have significant consequences for reducing the need for chemical plant protection products in fruit crops and the reduction of future insecticide resistance^59,60^ .

The impact of such claddings on natural enemies^49^ and pollinators^61^, which are key to the production of many fruit crops, requires further investigation. The commercially produced biological control agents *Orius laevigatus* and *Amblyseius swirskii* exhibited reduced dispersal rate under and a preference for the lower UV environment, respectively^49^. In addition, impact of these claddings on other fruit types is recommended as they may yield different responses.

## Methods

D. suzukii *rearing and colony maintenance.*

*D. suzukii* used in all experiments were obtained from a laboratory colony originating from raspberries collected in Trento, Italy in 2013. The flies were held in cages (32.5 cm × 32.5 cm × 32.5 cm; Bug-dorm, MegaView Science, Taichung, Taiwan), stored in climate chambers at 23 °C, 65 ± 5% relative humidity, 10 klux light intensity, and a photoperiod of 16 h light/8 h dark (LD 16/8). Adult *D. suzukii* were supplied with Drosophila Quick Mix Medium (Blades Biological Ltd, Cowden, Kent, UK) for oviposition and food, and supplemented with defrosted strawberry fruits.

### Laboratory choice tests

**Figure 6:**
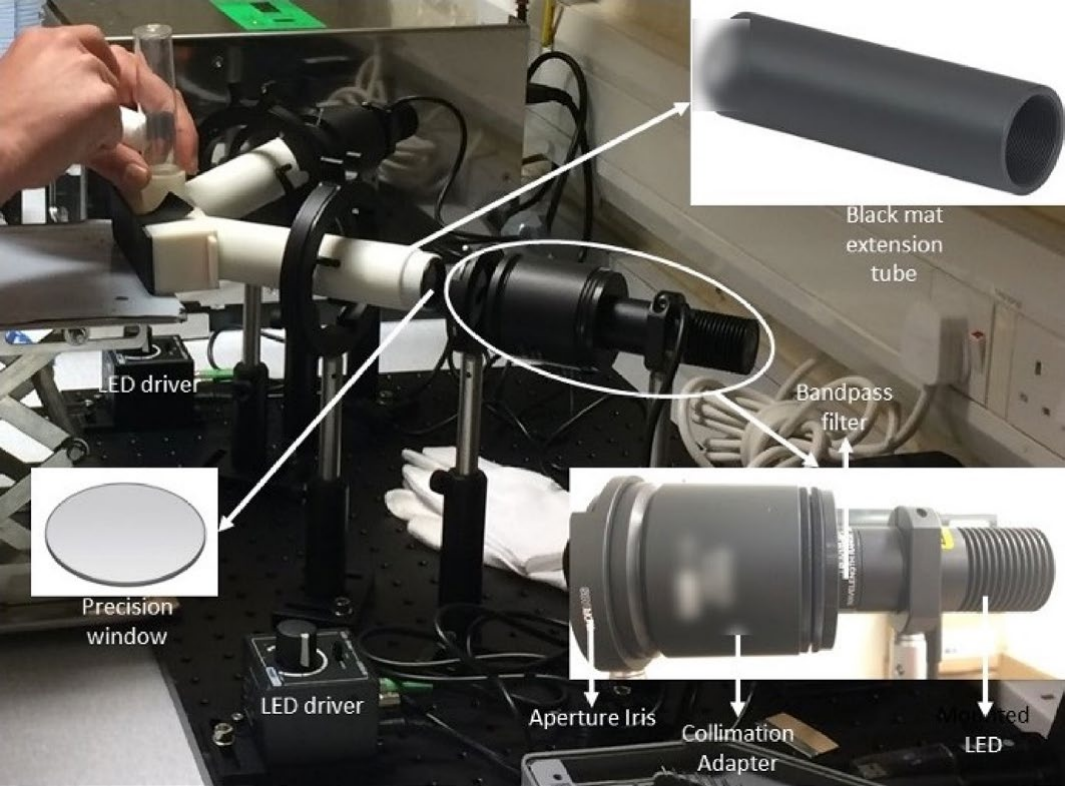
Y-maze optical setup.

The attractiveness of 9 wavelengths isolated in dark environment were assayed in binary choice "Y-maze"^62^ tests using 6–12 days old female *D. suzukii*. Light attraction experiments were conducted between 13 Dec 2017 and 17 Jan 2018 in a laboratory at NIAB EMR. A 3-D printed Y-maze with a central release chamber (length 116 mm, height 30 mm, depth 34 mm) and two arms with the light sources at the distal ends was constructed. The arms were black anodised aluminium beam tubes (THORLABS, SM1E60, outer diameter 30.5 mm) to minimise light reflection inside the arms. Access to the arms was blocked by use of UV-fused silica windows to prevent flies leaving Y-maze arms. The LED light wavelengths ranged between 340 and 660 nm. LEDs were fitted with a collimation adapter to ensure a uniform light beam was illuminated inside the Y-maze arms. The light beam was controlled by an aperture iris. Since the actual wavelength of LED light sources had a bandwidth of ± 10–30 nm (depending on the light source) bandpass filters were used to reduce the bandwidth and ensure the output wavelength was as close as possible to the nominal wavelength as shown in Table 2. Figure 6 shows the experimental optical Y-maze apparatus.

**Table 2:** Light-emitting diodes used in the laboratory choice experiment with details on wavelength, bandpass filters types (a Centre Wavelength, b Full Width Half Max) and collimation adapter.

THORLABS LED	Nominal LED Wavelength (nm)	(a) THORLABS Bandpass filter (nm)	(b) CWLa (nm)
M340L4	340	FB340-10	340 ± 2
M365L2	365	FB360-10	360 ± 2
M405L4	405	FB405-10	405 ± 2
M430L4	430	FB430-10	430 ± 2
M455L3	455	FL457.9-10	457.9 ± 2
M505L3	505	FL508.5-10	508.5 ± 2
M565L3	565	FB570-10	570 ± 2
M617L4	617	FB620-10	620 ± 2
M660L4	660	FB660-10	660 ± 2

The wavelength range of all lamps was 350–700 nm (FWHMb nm 10 ± 2) and all were used with a THORLABS collimation adapter (COP1-A).

The apparatus was tested for bias within the Y-maze choice test and it was shown that where identical light treatments were applied to both arms, *D. suzukii* females had no significant preferences.

All of the optical setup apparatus was fixed to an aluminium breadboard (450 mm × 600 mm × 12.7 mm). Sixty to seventy female *D. suzukii* (6 -12 days old) were transferred into the release chamber. Only females were used as these are responsible for fruit damage and, therefore, the focus of behavioural disruption. Subsequently the gates were opened simultaneously, and the flies were exposed to the light/dark conditions. After 30 min the gates were closed and the number of flies in each arm and the release chamber was counted and proportions determined. All experiments were carried out at 21 ± 1 °C and in a dark room to prevent interference with artificial and/or natural light. The light intensity in each arm was measured by an Ocean-FX spectrometer and set to 10 *µmolm*^*−*2^* s*^*−*1^ using LED controllers to ensure flies were exposed to equal light photon flux density in both arms for all tests.

Each test was repeated 6 times and approximately 60 flies were used in each test. The tests were classified into 3 groups (Table 3). The first group, control was to ensure there was no bias between the arms of the equipment. The second group tested light vs dark (phototaxis^18^) to ensure that *D. suzukii* responded to the different wavelengths in the absence of light in the other arm. Finally, the wavelengths were tested against 365 nm for preference to orientate (differential phototaxis^18^). 365 nm was chosen with prior knowledge of *D. melanogaster* sensitivities to blue and UV^21,22^ and the availability of LED’s at appropriate wavelengths.

**Table 3:** Wavelength tests on *D. suzukii* in the laboratory Y-maze experiments.

Control test	Light vs. Dark	Wavelengths vs. 365 nm
Dark vs. Dark	340 nm vs. Dark	365 nm vs. 340 nm
340 nm vs. 340 nm	365 nm vs. Dark	365 nm vs. 405 nm
365 nm vs. 365 nm	405 nm vs. Dark	365 nm vs. 430 nm
405 nm vs. 405 nm	430 nm vs. Dark	365 nm vs. 455 nm
430 nm vs. 430 nm	455 nm vs. Dark	365 nm vs. 505 nm
455 nm vs. 455 nm	505 nm vs. Dark	365 nm vs. 565 nm
505 nm vs. 505 nm	565 nm vs. Dark	365 nm vs. 617 nm
565 nm vs. 565 nm	617 nm vs. Dark	365 nm vs. 660 nm
617 nm vs. 617 nm	660 nm vs. Dark	–
660 nm vs. 660 nm	–	–

### Semi-field "no-choice" fruit damage experiments

The field trials in 2016 to 2018 were located at NIAB EMR, East Malling, Kent, UK (‘Ditton Rough’, N 51.289148, E 0.455042). The average temperature during each experiment was 11.2 °C, 11.4 °C and 11.5 °C and annual precipitation 578 mm, 470 mm and 598 mm in 2017, 2017 and 2018, respectively (Agrii weather station, East Malling, Kent, UK, N 51.287629, E 0.448587). Twelve tunnels (12 m × 2 m × 2.1 m; Knowle Nets Ltd, Bridport, Dorset, UK) were covered with insect mesh (1 mm × 1 mm, Knowle Nets Ltd, Bridport, Dorset, UK) with cladding materials (treatments) over the top. Test films were cut off at 30 cm above the ground to provide ventilation to the strawberry plants in the tunnels. The tunnels were orientated in north–south direction. In 2016, two UV-blocking films with different UV light transmission levels were tested. The films were coded UVopen and UV350 in a clear and diffuse version; there were three replicates per treatment. The following year four clear films were tested; UVopen, UV350, UV370, and UV400 with three replicates. Then in 2018 the 12 existing tunnels were divided by fine mesh (Dunelm Ltd., Syston, Leicestershire, UK) into two compartments and three films were compared; UVopen, UV370, and Lumitec, resulting in eight replicates of each (Table 4).

**Table 4:** Overview of the number of replicates used in the field tunnel trials in each year. * = not tested in that year. See (Fig. 2) for wavelength and transmission measurement.

Treatment	Film	Film code	2016	2017	2018
UVopen	Clear	No blocking	3	3	8
	Diffuse	No blocking	3	*	*
UV350	Clear	Up to 350 nm	3	3	*
	Diffuse	Up to 350 nm	3	*	*
UV370	Clear	Up to 370 nm	*	3	8
UV400	Clear	Up to 400 nm	*	3	*
Lumitec	Clear	Up to 430 nm	*	*	8

The light transmission of the experimental polyethylene film was measured from 300 to 2,500 nm at 2 nm steps using an Agilent Cary 7,000 Universal Measurement Spectrophotometer equipped with a diffuse reflectance accessory; an integrating sphere to capture all scattered light post transmission. Transmissions were measured on a 2 × 2 cm of film samples with the beam incident angle at 90°. Spectral transmissions of each of the materials are shown in Fig. 2.

All trials were conducted on everbearer strawberry plants, cv. Finesse (2016, 2018), and a commercially confidential proprietary cultivar (2017, 2018).

Bare root plants or plug plants (cv. Finesse and proprietary cultivar, respectively) were planted in 1 m peat bags. Bags were arranged end-to-end in the tunnels in one (2016 and 2017) or two rows (2018). In 2018, both cultivars were in all tunnels (10 bags of each, side-by-side in a row). Fertigation (fertilizer plus irrigation) was set to commercial standard and pesticides for disease control were applied only if necessary. At pink fruit stage (BBCH 81 – 85)^63^, known numbers of *D. suzukii* males and females were released into the tunnels (Table 5). In 2016 and 2017, air temperature and relative humidity was recorded in one tunnel of each treatment using a data logger (EL-USB-2; Lascar Electronics Ltd, Whiteparish, Wiltshire, UK). In 2018, a data logger (Tinytag plus 2 TGP-4500; Gemini Data Loggers Ltd, Chichester, West Sussex, UK) in each tunnel recorded air temperature and relative humidity.

**Table 5:** Trial period, tested strawberry variety, and *D. suzukii* inoculation details for each year in the field tunnelled experiments.

Year	Trial period	Variety	Date	No. *D. suzukii* introduced
2016	4 July–14 Oct	cv. Finesse	01/08	20♀ + 10♂
			09/09	10♀ + 5♂
2017	16 May–2 July	Proprietary cultivar	09/06	20♀ + 10♂
			20/06	40♀ + 20♂
2018	17 Apr–28 June	Proprietary cultivar and cv. Finesse	18/05	10♀ + 5♂
			24/05	20♀ + 10♂

During each trial period, where possible and where available, samples of 20 ripening and ripe fruits from the tunnels were collected every two weeks. Sampled fruits were weighed and incubated in clear Perspex boxes (20 × 10 × 10 cm) with ventilated lids at 22 ± 2 °C for three weeks. Once each week, emerging adult flies were removed from the boxes, and numbers of emerging *D. suzukii* counted under a light microscope (Leica MZ 8, Leica Biosystems GmbH, Nussloch, Germany). The degree of infestation was recorded as numbers of *D. suzukii* emerging per fruit mass (gram).

### Cloche choice experiments

To investigate the oviposition of *D. suzukii* under different light conditions the UVopen cladding, the Lumitec, and a dark (light excluded) were compared using outdoor 2-compartment choice chamber units^64^. Modified garden cloches (1 m × 0.45 m × 0.35 m; Tildenet Group Ltd, Bristol, UK) were fitted with the films and connected with a central release chamber. The release chamber was a10 cm diameter white PVC-cross (Spears Manufacturing Company, Sylmar, CA, US). During the experiments two cloches were connected on opposite sides of the release chamber with the remaining two sockets sealed. The first experiment was carried out from 4 September to 3 October 2018. Twenty female *D. suzukii* were released into the central chamber. Five blueberries cv. Duke were placed in a Petri dish in each cloche and the flies were left to oviposit for 24 h. Blueberries were used instead of strawberries because it is easier, and therefore more accurate, to observe eggs in these fruits. Subsequently, eggs in each fruit were counted under a light microscope. All film treatments were successively tested against each other. Each combination was repeated ten times. In a second experiment, eight releases of 50 female *D. suzukii* were done between 22 October and 3 November 2018. All treatments were simultaneously compared to one-another. Instead of blueberries, yellow sticky traps were used to trap *D. suzukii* adult females orientating into each cloche arm. This omitted any effect of fruit volatile compounds attracting flies and relied upon visual orientation.

## Statistical analysis

Data from the laboratory experiment was used to calculate Attraction Index (AI) which is the number of times a light treatment on one arm of the Y-maze was more attractive to flies compared to the light treatments^65^. AI = (N1—N2)/ (N1 + N2) where N1 and N2 are the number of the flies which have chosen arms 1 or 2, respectively. Then binominal distribution with Null hypothesis was used (equal distribution (= 0.5) between Y-maze arms). For this the number of flies (left and right) from all test replicates (6 reps per test) were summed and the values tested whether the sample was significantly (P < 0.05) different from the control.

The data from semi-field trials (numbers per gram of strawberry in each compartment at each sampling date), from each year, were analysed using repeated measures analysis of variance (ANOVA) in Genstat 13 (VSN International Ltd., 2010). When the assumptions of ANOVA were not met, data were square root transformed before analysis. However, reported means are from untransformed data for presentation purposes. The differences between means were compared using Fisher’s least significant difference (LSD) test at the 5% confidence level. The combined data set of the three consecutive years was analysed using restricted maximum likelihood (REML) in Genstat 13 (VSN International Ltd., 2010). The diffuse versions of the UVopen and UV350 films were excluded from the analysis as they were only tested in 2016. The differences between predicted means were compared using Tukey’s honest significant difference (HSD) test at the 5% confidence level.

The data from both cloche trials (numbers of eggs laid and numbers of *D. suzukii* on sticky traps) were analysed using a two-sample paired t-test in Genstat 13 (VSN International Ltd., 2010). When the assumptions of normality were not met, data was square root transformed before analysis. However, reported means are from untransformed data for presentation purposes. Means were compared using Tukey’s honest significant difference (HSD) test at the 5% confidence level.

## References

[1] Asplen MK (2015). Invasion biology of spotted wing drosophila (*Drosophila suzukii*): a global perspective and future priorities. J. Pest Sci..

[2] Lee JC (2011). In focus: spotted wing drosophila, *Drosophila suzukii*, across perspectives. Pest Manag. Sci..

[3] Bolda MP, Goodhue RE, Zalom FG (2010). Spotted wing drosophila: potential economic impact of a newly established pest. Agric. Resour. Econ. Updat..

[4] Haviland DR, Beers EH (2012). Chemical control programs for *Drosophila suzukii* that comply with international limitations on pesticide residues for exported sweet cherries. J. Integr. Pest Manag..

[5] Van Timmeren S, Mota-Sanchez D, Wise JC, Isaacs R (2018). Baseline susceptibility of spotted wing drosophila (*Drosophila suzukii*) to four key insecticide classes. Pest Manag. Sci..

[6] Gress BE, Zalom FG (2019). Identification and risk assessment of spinosad resistance in a California population of *Drosophila suzukii*. Pest Manag. Sci..

[7] Bale, J., Van Lenteren, J. & Bigler, F. Biological control and sustainable food production. *Philos. Trans. R. Soc. B: Biol. Sci.* **363**, 761–776 (2008). 10.1098/rstb.2007.2182 PMC2610108 17827110

[8] Iglesias LE, Nyoike TW, Liburd OE (2014). Effect of trap design, bait type, and age on captures of *Drosophila suzukii* (Diptera: Drosophilidae) in berry crops. J. Econ. Entomol..

[9] Tonina L (2018). Comparison of attractants for monitoring *Drosophila suzukii* in sweet cherry orchards in Italy. J. Appl. Entomol..

[10] Rajapakse NC, Kelly JW (1992). Regulation of chrysanthemum growth by spectral filters. J. Am. Soc. for Hortic. Sci..

[11] Van Haeringen C (1998). The development of solid spectral filters for the regulation of plant growth. Photochem. Photobiol..

[12] West J (2000). Spectral filters for the control of *Botrytis cinerea*. Ann. Appl. Biol..

[13] Antignus Y, Mor N, Ben Joseph R, Lapidot M, Cohen S (1996). Ultraviolet-absorbing plastic sheets protect crops from insect pests and from virus diseases vectored by insects. Environ. Entomol..

[14] Fennell, J. T., Fountain, M. T. & Paul, N. D. Direct effects of protective cladding material on insect pests in crops. *Crop. Prot.* (2019).

[15] Doukas D, Payne C (2007). The use of ultraviolet-blocking films in insect pest management in the UK; effects on naturally occurring arthropod pest and natural enemy populations in a protected cucumber crop. Ann. Appl. Biol..

[16] Solaiman, A. H. M., Nishizawa, T., Arefin, S. A., Sarkar, M. D. & Shahjahan, M. Effect of partially UV-blocking films on the growth, yield, pigmentation, and insect control of red amaranth (*Amaranthus tricolor*). *Curr. J. Appl. Sci. Technol.* 1–11 (2016).

[17] Hardie, R. C. Functional organization of the fly retina. In *Progress in Sensory Physiology*, 1–79 (Springer, New York, 1985).

[18] Schnaitmann C, Pagni M, Reiff DF (2020). Color vision in insects: insights from drosophila. J. Comp. Physiol. A.

[19] Schnaitmann C, Garbers C, Wachtler T, Tanimoto H (2013). Color discrimination with broadband photoreceptors. Curr. Biol..

[20] Wardill TJ (2012). Multiple spectral inputs improve motion discrimination in the drosophila visual system. Science.

[21] Schümperli RA (1973). Evidence for colour vision in *Drosophila melanogaster* through spontaneous phototactic choice behaviour. J. Comp. Physiol. A.

[22] Bernard GD, Stavenga DG (1979). Spectral sensitivities of retinular cells measured in intact, living flies by an optical method. J. Comp. Physiol..

[23] Hardie RC (2012). Polarization vision: Drosophila enters the arena. Curr. Biol..

[24] Zhu EY, Guntur AR, He R, Stern U, Yang C-H (2014). Egg-laying demand induces aversion of UV light in drosophila females. Curr. Biol..

[25] Kane EA (2013). Sensorimotor structure of drosophila larva phototaxis. Proc. Natl. Acad. Sci..

[26] Kelber A, Henze MJ (2013). Colour vision: parallel pathways intersect in drosophila. Curr. Biol..

[27] Rice KB, Short BD, Jones SK, Leskey TC (2016). Behavioral responses of *Drosophila suzukii* (Diptera: Drosophilidae) to visual stimuli under laboratory, semifield, and field conditions. Environ. Entomol..

[28] Kirkpatrick D, McGhee P, Hermann S, Gut L, Miller J (2016). Alightment of spotted wing drosophila (Diptera: Drosophilidae) on odorless disks varying in color. Environ. Entomol..

[29] Little CM, Chapman TW, Hillier NK (2018). Effect of color and contrast of highbush blueberries to host-finding behavior by drosophila suzukii (Diptera: Drosophilidae). Environ. Entomol..

[30] Little CM, Rizzato AR, Charbonneau L, Chapman T, Hillier NK (2019). Color preference of the spotted wing drosophila. *Drosophila suzukii*. Sci. Rep..

[31] Yamaguchi S, Wolf R, Desplan C, Heisenberg M (2008). Motion vision is independent of color in drosophila. Proc. Natl. Acad. Sci..

[32] Paulk A, Millard SS, van Swinderen B (2013). Vision in drosophila: seeing the world through a model’s eyes. Annu. Rev. Entomol..

[33] Humberg T-H, Sprecher SG (2017). Age-and wavelength-dependency of drosophila larval phototaxis and behavioral responses to natural lighting conditions. Front. Behav. Neurosci..

[34] Cronin TW, Bok MJ (2016). Photoreception and vision in the ultraviolet. J. Exp. Biol..

[35] Stone, T., Mangan, M., Ardin, P., Webb, B. *et al.* Sky segmentation with ultraviolet images can be used for navigation. In *Robotics: Science and Systems* (2014).

[36] Kirk R, Cielniak G, Mangan M (2020). L* a* b* fruits: A rapid and robust outdoor fruit detection system combining bio-inspired features with one-stage deep learning networks. Sensors.

[37] Clymans R (2019). Olfactory preference of *Drosophila suzukii* shifts between fruit and fermentation cues over the season: effects of physiological status. Insects.

[38] Keesey IW, Knaden M, Hansson BS (2015). Olfactory specialization in *Drosophila suzukii* supports an ecological shift in host preference from rotten to fresh fruit. J. Chem. Ecol..

[39] Kumar P, Poehling H-M (2006). UV-blocking plastic films and nets influence vectors and virus transmission on greenhouse tomatoes in the humid tropics. Environ. Entomol..

[40] Legarrea S, Karnieli A, Fereres A, Weintraub PG (2010). Comparison of UV-absorbing nets in pepper crops: Spectral properties, effects on plants and pest control. Photochem. Photobiol..

[41] Costa HS, Robb KL (1999). Effects of ultraviolet-absorbing greenhouse plastic films on flight behavior of *Bemisia argentifolii* (homoptera: Aleyrodidae) and *Frankliniella occidentalis* (Thysanoptera: Thripidae). J. Econ. Entomol..

[42] Chyzik R, Dobrinin S, Antignus Y (2003). Effect of a UV-deficient environment on the biology and flight activity of *Myzus persicae* and its hymenopterous parasite *Aphidius matricariae*. Phytoparasitica.

[43] Costa H, Robb K, Wilen C (2002). Field trials measuring the effects of ultraviolet-absorbing greenhouse plastic films on insect populations. J. Econ. Entomol..

[44] Dáder B, Gwynn-Jones D, Moreno A, Winters A, Fereres A (2014). Impact of uv-a radiation on the performance of aphids and whiteflies and on the leaf chemistry of their host plants. J. Photochem. Photobiol. B: Biol..

[45] Díaz BM, Biurrún R, Moreno A, Nebreda M, Fereres A (2006). Impact of ultraviolet-blocking plastic films on insect vectors of virus diseases infesting crisp lettuce. HortScience.

[46] Kuhlmann F, Müller C (2009). Development-dependent effects of UV radiation exposure on broccoli plants and interactions with herbivorous insects. Environ. Exp. Bot..

[47] Paul ND (2012). Ecological responses to UV radiation: interactions between the biological effects of UV on plants and on associated organisms. Physiol. Plantarum.

[48] Sal, J. *et al.* Influence of UV-absorbing nets in the population of *Macrosiphum euphorbiae* Thomas (Homoptera: Aphididae) and the parasitoid *Aphidius ervi* (Haliday) (Hymenoptera: Aphidiidae) in lettuce crops. In *Proceedings of Third International Symposium Biological Control Arthropods, *Christ Church, New Zealand, 329–337 (2009).

[49] Legarrea S, Weintraub P, Plaza M, Viñuela E, Fereres A (2012). Dispersal of aphids, whiteflies and their natural enemies under photoselective nets. Biocontrol.

[50] Legarrea S (2012). Dynamics of nonpersistent aphid-borne viruses in lettuce crops covered with UV-absorbing nets. Virus Res..

[51] Legarrea S (2012). Diminished uv radiation reduces the spread and population density of *Macrosiphum euphorbiae* (Thomas) [Hemiptera: Aphididae] in lettuce crops. Hortic. Sci..

[52] Dáder B, Moreno A, Gwynn-Jones D, Winters A, Fereres A (2017). Aphid orientation and performance in glasshouses under different UV-a/UV-b radiation regimes. Entomol. Exp. et Appl..

[53] El-Aal HAA, Rizk AM, Mousa IE (2018). Evaluation of new greenhouse covers with modified light regime to control cotton aphid and cucumber (*Cucumis sativus* L.) productivity. Crop. Prot..

[54] Kigathi R, Poehling H-M (2012). UV-absorbing films and nets affect the dispersal of western flower thrips, *Frankliniella occidentalis* (Thysanoptera: Thripidae). J. Appl. Entomol..

[55] Bueno, E. *et al.* Response of wild spotted wing drosophila *(Drosophila suzukii)* to microbial volatiles. *J. Chem. Ecol.* 1–11 (2019). 10.1007/s10886-019-01139-4 31879864

[56] Renkema JM, Buitenhuis R, Hallett RH (2017). Reduced *Drosophila suzukii* infestation in berries using deterrent compounds and laminate polymer flakes. Insects.

[57] Erland LA, Rheault MR, Mahmoud SS (2015). Insecticidal and oviposition deterrent effects of essential oils and their constituents against the invasive pest *Drosophila suzukii* (Matsumura) (Diptera: Drosophilidae). Crop. Prot..

[58] Wallingford AK, Cha DH, Loeb GM (2018). Evaluating a push–pull strategy for management of *Drosophila suzukii Matsumura* in red raspberry. Pest Manag. Sci..

[59] Smirle MJ, Zurowski CL, Ayyanath M-M, Scott IM, MacKenzie KE (2017). Laboratory studies of insecticide efficacy and resistance in *Drosophila suzukii* (Matsumura) (Diptera: Drosophilidae) populations from British Columbia, Canada. Pest Manag. Sci..

[60] Shaw B, Brain P, Wijnen H, Fountain MT (2019). Implications of sub-lethal rates of insecticides and daily time of application on *Drosophila suzukii* lifecycle. Crop. Prot..

[61] van der Blom J (2010). Applied entomology in spanish greenhouse horticulture. Proc. Neth. Entomol. Soc. Meet.

[62] Fingerman M, Brown FA (1952). A "purkinje shift" in insect vision. Science.

[63] Meier, U. Phenological growth stages. In *Phenology: An Integrative Environmental Science*, 269–283 (Springer, New York, 2003).

[64] Doukas D, Payne CC (2014). Greenhouse whitefly (Homoptera: Aleyrodidae) dispersal under different UV-light environments. J. Econ. Entomol..

[65] Palanca, L., Gaskett, A. C., Günther, C. S., Newcomb, R. D. & Goddard, M. R. Quantifying variation in the ability of yeasts to attract *Drosophila melanogaster*. *PLoS ONE* **8** (2013). 10.1371/journal.pone.0075332 PMC3783394 24086510

